# Symmetry breaking in core-valence double ionisation of allene

**DOI:** 10.1038/s42004-023-00934-1

**Published:** 2023-07-03

**Authors:** Veronica Ideböhn, Roberto Linguerri, Lucas M. Cornetta, Emelie Olsson, Måns Wallner, Richard J. Squibb, Rafael C. Couto, Leif Karlsson, Gunnar Nyman, Majdi Hochlaf, John H. D. Eland, Hans Ågren, Raimund Feifel

**Affiliations:** 1 University of Gothenburg, Department of Physics, Origovgen 6B, SE-412 58 Gothenburg, Sweden; 2 Universite Gustave Eiffel, COSYS/IMSE, 5 Bd Descartes 77454, Champs sur Marne, France; 3 Uppsala University, Department of Physics and Astronomy, Box 516, SE-751 20 Uppsala, Sweden; 4 Department of Applied Physics, Gleb Wataghin Institute of Physics, State University of Campinas, Campinas, Brazil; 5 Division of Theoretical Chemistry and Biology, School of Engineering Sciences in Chemistry, Biotechnology and Health, KTH Royal Institute of Technology, SE-106 91 Stockholm, Sweden; 6 University of Gothenburg, Department of Chemistry and Molecular Biology, Kemigården 4, SE-412 96 Gothenburg, Sweden; 7 Oxford University, Department of Chemistry, Physical and Theoretical Chemistry Laboratory, South Parks Road, Oxford, OX1 3QZ UK

**Keywords:** Chemical physics, Computational chemistry

## Abstract

Conventional electron spectroscopy is an established one-electron-at-the-time method for revealing the electronic structure and dynamics of either valence or inner shell ionized systems. By combining an electron-electron coincidence technique with the use of soft X-radiation we have measured a double ionisation spectrum of the allene molecule in which one electron is removed from a C1s core orbital and one from a valence orbital, well beyond Siegbahns Electron-Spectroscopy-for-Chemical-Analysis method. This core-valence double ionisation spectrum shows the effect of symmetry breaking in an extraordinary way, when the core electron is ejected from one of the two outer carbon atoms. To explain the spectrum we present a new theoretical approach combining the benefits of a full self-consistent field approach with those of perturbation methods and multi-configurational techniques, thus establishing a powerful tool to reveal molecular orbital symmetry breaking on such an organic molecule, going beyond Löwdins standard definition of electron correlation.

## Abstract (web summary)

Allene serves as a model to study multiple ionization of organic molecules. Here, the authors use synchrotron radiation-based multi-particle coincidence techniques and high-level ab initio calculations to propose a simple physical model to elucidate the symmetry breaking in core-valence double ionization of allene.

## Introduction

Allene or propadiene (C_3_H_4_), which in its neutral form belongs to the D_2*d*_ symmetry point group, is a highly interesting molecule, not only from a fundamental point of view, but also because of its putative relevance in astrophysical contexts where high energy photons are present. Early on, its single ionisation was examined by photoionisation mass spectrometry^1^, photoelectron spectroscopy^2–5^ and photoelectron-photoion coincidence spectroscopy^6^. Subsequently, its double and triple ionisation have been studied by charge exchange methods accompanied by theoretical calculations^7,8^, where the lowest vertical double ionisation energy to the expected ground-state triplet was determined as 28.20.3 eV^7^. In a more recent work^9^, double and triple ionisation of allene using single-photon excitation were investigated in greater details by means of a complementary set of electron-electron, electron-ion and ion-ion coincidence spectroscopy methods, focusing on final states which reflected the removal of two or three electrons from the valence shell.

In an alternative high-energy form of double ionisation, which the present work focuses on, one of the two electrons is removed from an inner shell and one from the valence shell, leading to the formation of rich molecular spectra showing core-valence doubly ionized states^10–12^. The states produced in core-valence double ionisation were previously denoted in the literature as core shake-off satellites; their existence has been known since the early days of core level photoelectron spectroscopy, where they were detected with a single channel technique of the Siegbahn type, i.e. without coincidences, and thus without measurement of their spectra. The doubly-charged cations formed in this way have short lifetimes and undergo Auger decay to produce triply (or more highly) valence-ionized final states, mostly the same states characterised in the aforementioned recent investigation on multiply-valence-ionized allene^9^.

Some of the first molecular studies of core-valence double ionisation using electron-electron coincidence detection^11,12^, revealed that core-valence spectra often resemble the corresponding single ionisation valence photoelectron spectra, suggesting the operation of an overall Coulomb interaction rather than a specific hole-hole coupling mechanism. Quantitatively different spectra are observed for different core charge localisations, i.e. when the core electron originates from different atoms in the same molecule. In several molecules consisting of substituent groups symmetrically bound to a central atom, choice of the central atom for removal of the core electron leads to core-valence spectra resembling the normal photoelectron spectra quite closely, but removal of a core electron from an outer substituent produces quite different core-valence spectra. This contrast is notable, for example, in the core-valence spectra of CF_4_, CCl_4_, SF_6_ and Si(CH_3_)_4_^13^. A similar effect in the core-valence spectra of SO_2_ has been attributed to a (pseudo-)Jahn-Teller effect^14^. Mimicry of the valence photoelectron spectrum by core-valence double ionisation spectra holds even in cases where the core levels are multiple because of spin-orbit splitting^12^.

**Fig. 1:**
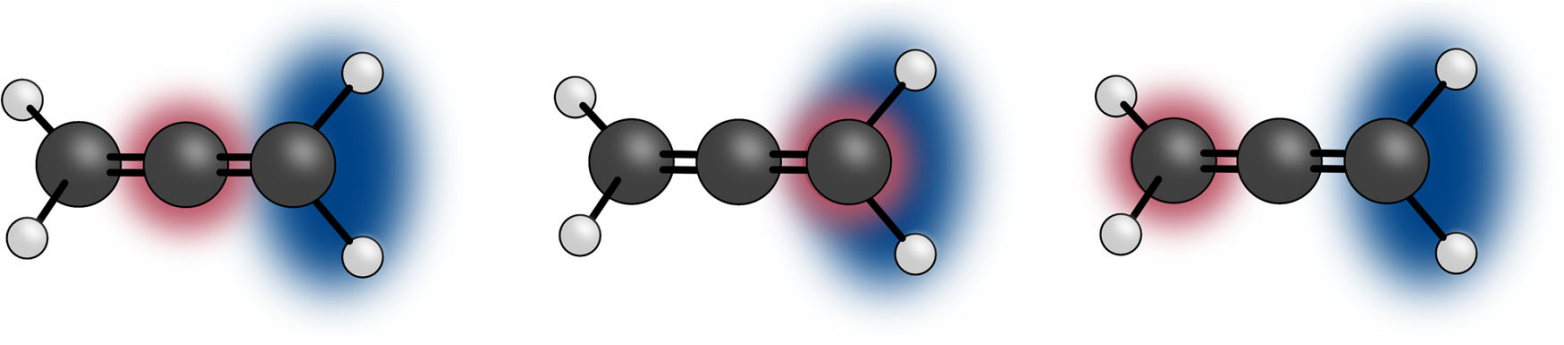
Core-hole localisation in allene. Schematic of the allene molecule illustrating the localisation of the different core hole possibilities (red) in relation to the valence ionisation part (blue).

In this work, we examine core-valence double ionisation of allene, schematically illustrated in a simplified picture shown in Fig. 1. The core-valence electron spectrum produced when a C1s electron is removed is qualitatively different from the valence photoelectron spectrum, as the band produced by ionisation from the outermost valence orbital appears as a triple feature rather than the expected single feature.

**Fig. 2:**
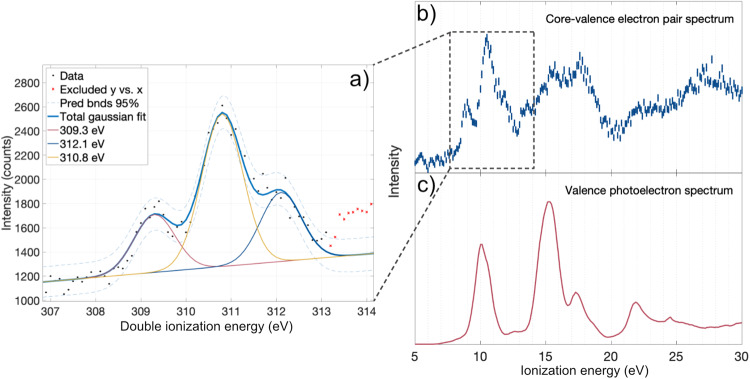
Electron spectra of valence and core-valence ionized allene. **a** A close-up of the lowest core-valence double ionisation feature obtained at 350.4 eV at the synchrotron radiation facility BESSY-II which comprises a central peak at 310.8 eV about twice as strong as the two adjacent peaks at 309.3 and 312.1 eV, respectively. The results of a least-square curve-fitting analysis are also included in this panel. **b** holds the core-valence double ionisation spectrum of allene from (**a**), but with shifted energy scale for comparison to single ionisation valence photoelectron spectrum of allene (**c**). **c** is from ionisation at 40.8 eV photon energy provided by a gas discharge lamp compared to the core-valence double ionisation spectrum of allene and is corrected for pre-pulse ionisation events.

The experimental data displayed in Fig. 2 were obtained at the synchrotron radiation facility BESSY-II of the Helmholtz Zentrum für Materialien in Berlin and in our laboratory at the University of Gothenburg as detailed in the Methods section below. The allene case is an extraordinary case; to the best of our knowledge, nothing comparable has been reported up to now in the literature, and in particular no such effect is seen in the core-valence spectra of the almost isoelectronic, symmetric CO_2_ molecule^13,15^. To interpret this phenomenon, we propose a simple physical model, and also invoke high level ab initio calculations as described in the Methods section below, going beyond Löwdins standard definition of electron correlation.

## Results

Panels (a) and (b) of Fig. 2 show the core-valence double ionisation spectrum obtained at a photon energy of 350.4 eV, where panel (b) is displayed on an energy scale shifted by 300.3 eV to make it comparable to the conventional (single ionisation) photoelectron spectrum of allene in panel (c) taken at 40.8 eV.

As can be seen, the core-valence spectrum looks qualitatively different, with the first core-valence band presenting a triple structure in comparison to a single feature in the corresponding region of the conventional valence photoelectron spectrum. This triple structure can be seen in greater detail as a close-up view in panel (a) of Fig. 2, now on an absolute double ionisation energy scale. From this figure, we can see that the central feature at about 310.8 eV is about twice as strong as the features to its left and right at about 309.3 and about 312.1 eV, respectively. A comparable figure for CO_2_ is shown for comparison in Supplementary Fig. 1.

**Fig. 3:**
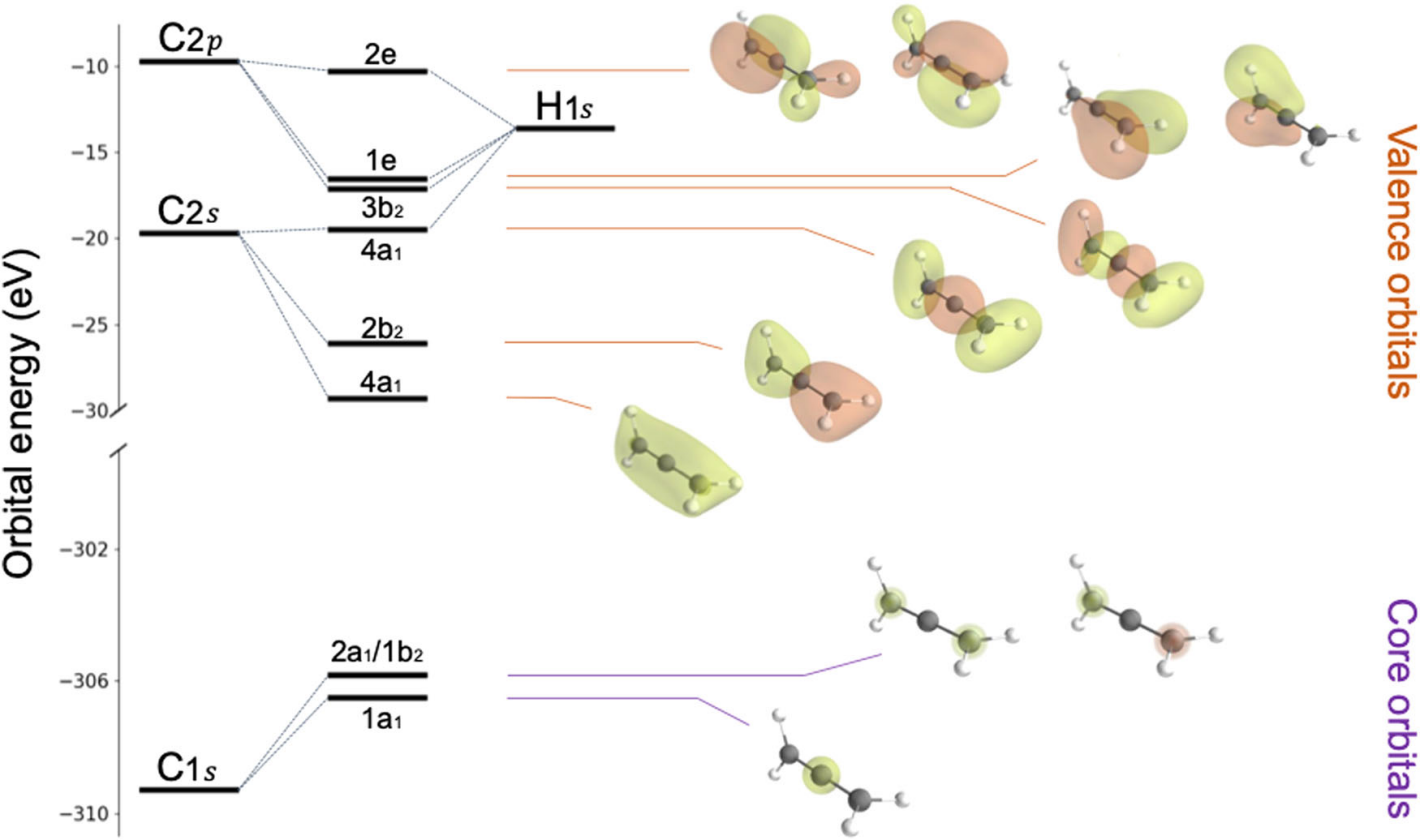
Energy levels and molecular orbitals of allene. Molecular energy diagram and iso-potential-energy-surfaces of the relevant orbitals. Purple energies represent core orbitals and orange energies represent valence orbitals. Ionisation from either core orbital will lead to localisation and symmetry breaking. The molecule is visualised in its neutral shape with *H*_2_ groups on each side perpendicular to each other.

To aid the analysis and interpretation of this finding, Fig. 3 displays a molecular orbital energy diagram including iso-potential-energy surfaces of the relevant orbitals.

We attribute this triple structure to breaking of the molecular symmetry by localisation of the core hole on just one of the two terminal C_*T*_ atoms. If the two orthogonal *π*-bond orbitals comprising the outermost 2e valence orbital are also considered as localised, removal of a valence electron from the *π*-bond containing the core-ionized C atom will result in a short distance between the positive charges, and a relatively high energy. In contrast, when a valence electron comes from the more distant *π*-bond the energy must be lower. If the core electron is removed from the central C_*C*_ atom both *π*-electron pairs are affected equally, and also a central peak can thus be expected. The conceptual justification for this simple model is that the double ionisation process takes place extremely rapidly, in a time of the order of 100 attoseconds for the electrons to leave the atomic environment. The two outer C1s orbitals are exactly degenerate and non-orthogonal, so their + and - combination orbitals have a small energy separation, but one whose associated exchange time h/*δ*E is much longer than the ionisation time. The treatment of these C1s orbitals as localised is thus fully justified. The case of the two *π*-bonds making up the 2e outer valence orbital is more involved; at the equilibrium molecular geometry, and neglecting vibronic interactions, they are degenerate but orthogonal, so have zero splitting into + and - combinations, so may be considered localised by the same criterion. In view of the broken symmetry and the attosecond timescale on which the core-valence process must take place, we note its relevance to pure electron-correlation-driven charge migration in ionized molecules as predicted more than 20 years ago by Cederbaum and coworkers^16^, which direct observation is currently hunted for in experimental investigations^17,18^.

To examine this simple explanation more thoroughly, we turn to ab-initio quantum chemical calculations. A theoretical approach to corevalence states faces a particular challenge, because the substantial relaxation following the creation of a core hole calls for a self-consistent field procedure, while valence ionisation is often better treated by perturbation theory and variational approaches. This is because the difference in electron correlation between the initial ground state and the final valence ionized state is relatively small as is the valence orbital relaxation energy. In fact, relaxation and correlation contributions (which are separable quantities assuming Löwdins definition of electron correlation) tend to counteract each other in the total energy, which is a prerequisite for Koopmans’ theorem^19^ to be applicable for modelling valence photoelectron spectra. Because core-valence double hole states are subject to strong electron relaxation upon the creation of the core hole, the final double hole state energies will depart from an extended Koopmans’ theorem where the transition energies are given by a sum of two (negative) orbital energies corrected by the hole-hole interaction.

An electronic structure core-valence approach should ideally be able to cope with these aspects of core and valence ionisation at the same time. Several earlier works on core-valence spectra have adopted multi-configurational self-consistent field theory within the complete active or restricted active space approaches, or by configuration-interaction (CI) techniques employing a fixed set of orbitals^11,12,20–22^. The latter are with preference optimised for the single core hole state, so that the major relaxation effect already is accounted for when the CI procedure is applied. The CI expansion coefficients can then be used as a guide for core-valence intensities, although the full core-valence cross-section also requires orbital transition moments with the double electronic continuum, which so far have not been evaluated.

In this work, another methodological approach is applied, which has the particular advantage of covering a large part of, or even reaching the full core-valence spectrum, namely restricted open-shell Hartree-Fock (ROHF), augmented by second-order perturbation theory (PT2). The ROHF scheme has the great advantage that it offers a well-defined independent particle interpretation of the spectrum with each core-valence state being defined by the smallest possible combination of determinants that fullfill spatial and spin symmetry requirements of the state. The correlation energy can so be defined (in the spirit of Löwdins original definition for single determinant states) as the difference between that energy and the full non-relativistic energy. For each core-valence double hole state of a neutral ground state molecule this leads to singlet states with a combination of two determinants and one-determinant triplet states, where the latter contains three degenerate spin-sublevels. Though spin or spin projection is not discriminated in the measurements, the separation of singlets and triplets for a given core-valence orbital configuration reflects the overlap and exchange of the participating core and valence orbitals (formally the sum of a Coulomb and an exchange integral). The energy separation thus reflects the localisation of the valence orbital with respect to the core orbital. The charge flow and screening relaxation of the core hole can here significantly alter the singlet-triplet splitting, something that is reflected by the different values of this splitting for the different valence orbitals, but also for the two different core holes, which are actually predicted. Coping for the possibility of having all possible spin projections for the two continuum electrons leads to the assumption that the spectral core-valence states are statistically populated with a 3/1 ratio for triplets to singlets.

Before analyzing the core-valence spectrum of allene it is instructive to analyze the corresponding valence photoelectron spectrum (see panel a) of the Supplementary Fig. 2) and the corresponding core level spectrum (see panel b) of the Supplementary Fig. 2), the spectra reflecting single valence and single core electron ionisation, respectively. As shown in Supplementary Note 2, we see that the advised ROHF level of theory covers the energy positions of all the 2e, 1e, 3b_2_, 4a_1_, 2b_2_ and 3a_1_ molecular orbital levels quite well, only marginally overstretching it, going from 9 to 27 eV. The corresponding Koopmans’ energies going from 10 to 29 eV seemingly match the onset of the spectrum perfectly but overstreches it by some eVs. As is well known from analysis of abundant valence photoelectron spectra, Koopmans’ theorem usually gives the correct ordering of molecular orbital levels as it tends to balance out relaxation and correlation corrections which are of different signs (the N_2_ and pyridine molecules being well-known exceptions). Thus ROHF, including relaxation but excluding correlation (in Löwdin’s definition) unravels the correlation correction, which may explain why the onset of the ROHF spectrum is 1 eV lower than the experimental spectrum while the Koopmans energy is spot on (there is one electron more to correlate in the ground than in the final state). The final state correlation effect may increase when going to deeper orbitals as structural, near-degeneracy effects then may become progressively more prevalent. As indicated by the basis set tests, discussed in the computational section below, the applied ANO-RCC-VQZP basis set gives transition energies close to the converged ROHF and PT2 values.

What concerns the core level ionisation spectrum, as shown in Supplementary Note 2, the ROHF (or so-called open-shell ΔSCF) values are only a few tenths of an eV away from the experimental values, with excellent reproduction of the chemical shift, at least as an energy difference of vertical transition energies visually extracted from the spectrum. The remaining error for the absolute ionisation energy is probably due to core-core correlation and a relativistic correction (ca. 0.2 eV), both giving a negative error to the ionisation energy, but which presumably is very chemically inert^23,24^. It is also instructive to note, that while Koopmans energies are off by more than 10 eV because relaxation is neglected, they largely maintain the prediction of the chemical shift between the two core ionisation potentials. This indicates that the shift here is a ground state charging effect while the final state contribution, the relaxation energy, provides roughly equal contribution to the shift.

**Fig. 4:**
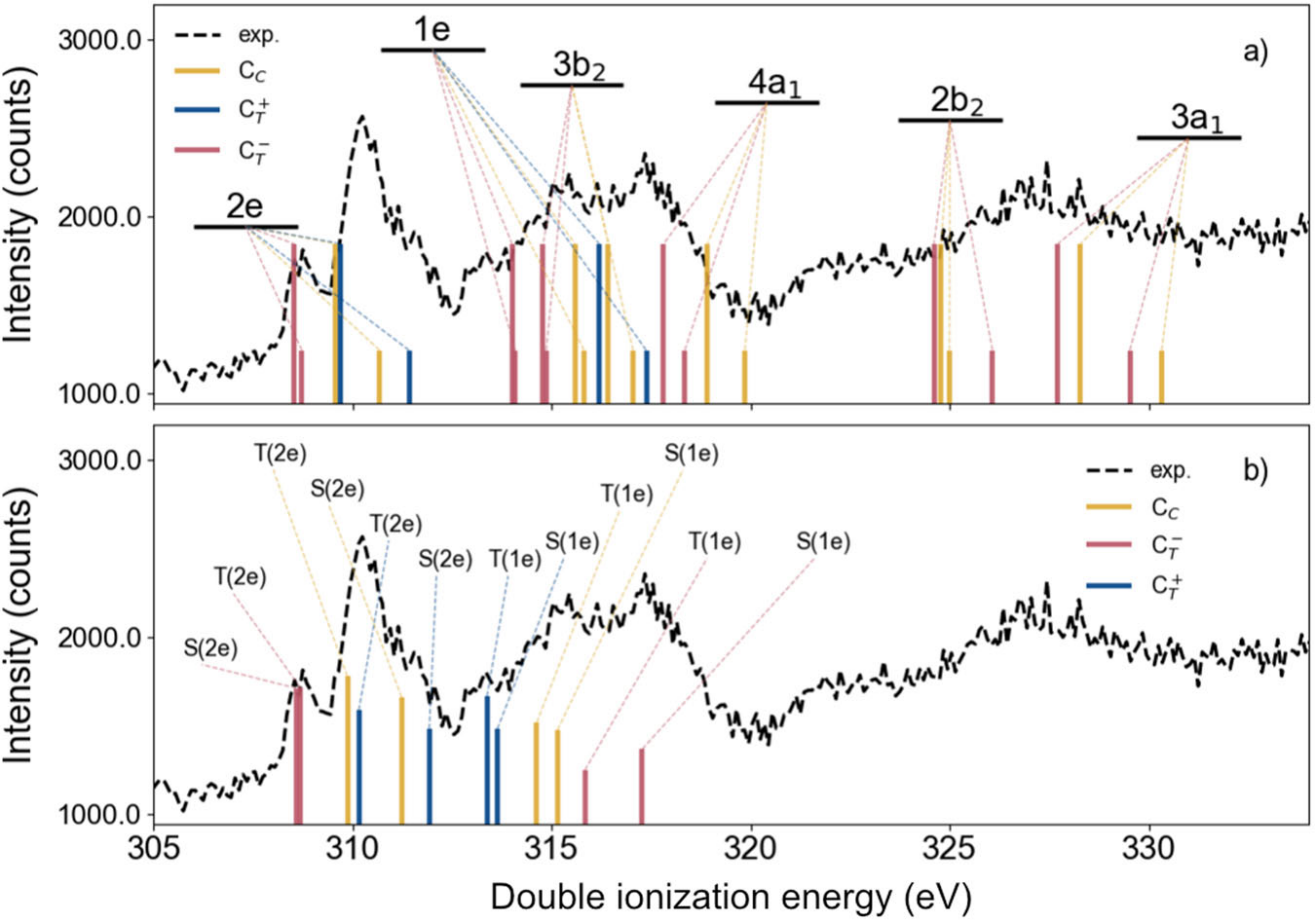
Theoretical versus experimental results of core-valence doubly ionized allene. Theoretical calculations of the core-valence double ionisation energies of allene, in comparison to the experimental data, based on the ROHF methodology (**a**) and CASCI (**b**). *C*_*C*_ and *C*_*T*_ denote the central and terminal carbon, respectively. In (**a**) the bars were multiplied by 1 and 3 for singlet and triplet states, respectively, while in (**b**) the bars were multiplied by the coefficients squared of the configuration state function related to the core-valence state in the CI expansion. In the latter case, only the 2*e* and 1*e* bands were studied and the spin multiplicities are indicated. The calculated CASCI transition energies (**b**) were uniformly shifted by 3.1 eV, while the ROHF transition energies (**a**) are unshifted.

The analysis of the core level and valence photoelectron spectra provides the basis for our analysis of the core-valence spectrum with the same method, which is displayed in Fig. 4. Energies of the different molecular orbitals assigned to the peaks in Fig. 4 are illustrated in Fig. 3 where the orbitals are visualised on a neutral allene molecule. The capability of ROHF (and Koopmans’ theorem) for assigning the valence photoelectron spectrum of allene as well as for its core level spectrum, with the proper relaxation included, can thus here be seen as a necessary condition for moving on to analyze the core-valence spectrum. Note that each orbital level known from the valence photoelectron spectrum is now quadrupled for a non-degenerate MO, with two spin couplings and two core holes while the outer degenerate e_1_ and e_2_ orbital levels are sextupled. As discussed above, we assign each triplet a threefold higher intensity compared with the corresponding singlet. We see that the distribution of ROHF energies represents the whole spectrum well, making it possible to assign broad groupings of energies to participating orbitals, although without interpretation of the fine structures. We note that the full core-valence spectrum is considerably broader than the valence photoelectron spectrum, going from 15 to 30 eV, owing to the effect of the more attractive doubly charged potential. There are also some gaps in the spectrum, notably the conspicuous one between 320 and 325 eV, where the absence of calculated ROHF structures is in agreement. We note that the singlet-triplet splittings show a great deal of variability with respect to the molecular orbitals and core hole, patterns reflecting different degrees of localisation and relaxation of the molecular orbitals with respect to the core hole.

Turning to the second-order perturbation theory results, see Table 1, we find that these contributions tend to lower the total core-valence transition energies for the outer levels, whereas they increase the energies for the more inner lying levels–this trend is, however, not completely consistent. These corrections are mostly well within an eV, and do not fill the above-mentioned 320–325 eV gap. To understand this observation we first note that the additional (dynamical) correlation of two extra electrons in the ground state can be weighed against the increased structural correlation as one moves upwards on the ionisation energy scale of the spectrum (deeper molecular orbitals). We recall that PT2 is a dynamical correlation method applied to single-reference, quasi-particle, states (or correlated one-particle states) and improves the energetics of such states compared to ROHF. The fact that it does not cover up for the energy gap between 320 and 325 eV in the allene core-valence spectrum leads to the speculation that the spectral structures in this gap originate in static correlation due to near-degeneracies leading to the so-called breakdown of the molecular orbital picture states well-known from single ionisation electron inner-valence spectra and states previously denoted in the literature as core-level shake-up satellites. Compared to valence photoelectron spectra, the additional core hole in the core valence process may increase the possibilities for internal and semi-internal configurational near-degeneracies and so promote these states also in parts with lower spectral energy, like here in the 320-325 eV region. This may also be the reason for the different signs of the PT2 corrections between upper and lower parts of the spectrum. Despite this shortcoming, single reference PT2 has the advantage of being applicable to the full core-valence spectrum and its routine application thus makes it an excellent complement to ROHF. The inclusion of CASCI calculations on 2e and 1e core-valence spectra largely enforce the ROHF–PT2 results in that part of the energy region. From the leading coefficients of the CASCI expansion, we could get a first estimate of the importance of the various configuration state functions in the spectra. The CASCI results make it possible to at least tentatively assign states with substructures of the well-separated first 2e band, while this is harder to achieve for the 1e transitions in the second band owing to the overlap with the 3b_2_ and 4a_1_ transitions.

**Table 1:** Numerical energies calculated at different levels of theory.

spec.	state	ANO-RCC-VQZP	ANO-RCC-VTZP	ANO-RCC-VDZP	CASCI^a^	CASSCF^b^
XPS	C_*C*_	290.90 (290.72)	291.23 (290.82)	292.40 (292.29)		
	C_*T*_	290.65 (290.10)	291.04 (290.33)	292.07 (291.77)		
UPS	2*e*	8.81 (10.35)	8.83 (10.26)	8.97 (10.10)		
	1*e*	14.88 (15.24)	14.90 (15.16)	15.07 (15.06)		
	3*b*_2_	16.61 (15.55)	16.64 (15.48)	17.85 (15.40)		
	4*a*_1_	18.81 (17.55)	18.84 (17.47)	19.00 (17.37)		
	2*b*_2_	24.16 (21.40)	24.18 (21.31)	24.29 (21.17)		
	3*a*_1_	27.44 (24.87)	27.45 (24.78)	27.59 (24.69)		
CV	C_*C*_(2*e*) T	309.56 (309.07)	309.97 (309.00)	310.66 (310.30)	309.86	308.0
	C_*C*_(2*e*) S	310.67 (310.56)	311.00 (310.03)	311.64 (311.35)	311.23	308.0
	C${}_{T}^{+}$(2*e*) T	309.70 (309.24)	311.01 (309.49)	311.62 (310.62)	310.15	309.2
	C${}_{T}^{+}$(2*e*) S	311.41 (311.97)	312.22 (310.86)	312.75 (312.02)	311.92	310.4
	C${}_{T}^{-}$(2*e*) T	308.53 (307.82)	309.02 (308.29)	309.62 (309.50)	308.67	308.7
	C${}_{T}^{-}$(2*e*) S	308.72 (307.86)	309.11 (308.45)	309.69 (309.54)	308.58	308.7
	C_*C*_(1*e*) T	315.58 (315.59)	315.62 (313.57)	316.33 (315.04)	314.62	313.0
	C_*C*_(1*e*) S	315.81 (315.82)	315.98 (314.00)	316.64 (315.36)	315.14	313.2
	C${}_{T}^{+}$(1*e*) T	316.19 (316.20)	316.42 (314.87)	317.07 (316.07)	313.37	314.2
	C${}_{T}^{+}$(1*e*) S	317.37 (317.38)	317.52 (316.01)	318.11 (317.17)	313.63	314.5
	C${}_{T}^{-}$(1*e*) T	314.00 (314.02)	314.67 (312.61)	315.30 (313.92)	315.83	313.3
	C${}_{T}^{-}$(1*e*) S	314.08 (314.09)	314.76 (312.82)	315.37 (314.00)	317.27	313.3
	C_*C*_(3*b*_2_) T	316.42 (318.17)	316.90 (316.21)	317.37 (317.12)		
	C_*C*_(3*b*_2_) S	317.04 (318.63)	317.61 (316.90)	317.84 (317.65)		
	C_*T*_(3*b*_2_) T	314.78 (315.68)	315.12 (314.57)	315.77 (315.70)		
	C_*T*_(3*b*_2_) S	314.86 (315.72)	315.17 (314.58)	315.82 (315.71)		
	C_*C*_(4*a*_1_) T	318.91 (320.66)	319.18 (318.51)	319.74 (319.69)		
	C_*C*_(4*a*_1_) S	319.85 (321.69)	319.79 (319.19)	320.30 (320.31)		
	C_*T*_(4*a*_1_) T	317.81 (320.06)	318.59 (318.37)	318.60 (318.84)		
	C_*T*_(4*a*_1_) S	318.34 (321.31)	319.14 (318.88)	318.95 (319.15)		
	C_*C*_(2*b*_2_) T	324.78 (326.61)	325.05 (323.17)	325.39 (324.32)		
	C_*C*_(2*b*_2_) S	324.99 (327.03)	325.27 (323.45)	325.54 (324.48)		
	C_*T*_(2*b*_2_) T	324.60 (325.03)	324.82 (323.03)	325.43 (323.86)		
	C_*T*_(2*b*_2_) S	326.07 (325.85)	325.59 (323.62)	326.28 (324.50)		
	C_*C*_(3*a*_1_) T	328.28 (328.71)	328.43 (326.26)	329.22 (327.83)		
	C_*C*_(3*a*_1_) S	330.32 (330.81)	330.86 (328.80)	331.53 (330.26)		
	C_*T*_(3*a*_1_) T	327.71 (327.39)	328.03 (326.67)	328.76 (326.97)		
	C_*T*_(3*a*_1_) S	329.52 (330.01)	330.16 (329.05)	330.76 (329.02)		

Transition energies calculated with ROHF wave functions without and with second-order perturbation theory PT2 (in parenthesis), in eV. For the core-valence (CV) states, the spin multiplicity is denoted by T (triplet) or S (singlet).

^a^CASCI calculations were performed together with the ANO-RCC-VQZP basis set and the results were shifted according to Fig. 4.

^b^CASSCF calculations were performed with the cc-pwCVQZ basis set and the results were shifted by 308.0 eV.

Examination of the computational results in Fig. 4 shows that at least some ideas of the simple model are supported. The lowest energy part of the triplet of broad bands arising from valence ionisation from the outermost orbital is attributed entirely to the core-hole on an outer carbon, giving two states with a small spin splitting, consistent with a long distance between the unpaired electrons. The central peak arises mainly from core ionisation at the central carbon atom, with a moderate spin splitting. The remaining part also has a contribution from a terminal carbon core ionisation with a larger spin splitting consistent with a short unpaired electron separation, which also explains the higher energy peak of the triplet. The simple model thus seems to work well along with the complementary quantum chemistry calculations—here ROHF, PT2 and CASCI—to get an overall assessment of the spectra, a finding that probably can be capitalised on when turning to other molecular core-valence spectra of molecules of the size of allene or larger. The symmetry breaking observed in this work can be expected to be a general phenomenon for CV spectra of polyatomic molecules containing core orbitals in symmetry positions. We refer here to analogy with XPS (single core hole) spectra of such molecules, where symmetry breaking has been analysed as a result of vibronic coupling between symmetry-delocalised core hole states over non-symmetric vibrational modes, thereby distorting the molecular frame away from the original symmetry.

In this work, we have examined the core-valence double ionisation spectrum of the allene molecule above the C1s edge. This showcase experimental spectrum is compared with a simple physical model and also with the results of quantum chemical calculations based on new theoretical approaches where the benefits of a full self-consistent field approach were combined with those of perturbational methods and of a configuration-interaction variational treatment, going beyond Löwdin’s standard definition of electron correlation. Our results suggest a clear effect of symmetry breaking when the core electron is ejected from one of the two outer carbon atoms of allene.

## Methods

### Experimental details

The experiments were conducted at the synchrotron radiation facility BESSY-II of the Helmholtz Zentrum für Materialen in Berlin and in our laboratory at the University of Gothenburg. The gaseous allene sample was obtained commercially with a stated purity of ≥95% and was let into the light-matter interaction chamber of our spectrometer in the form of an effusive beam. As the sample is ionized, a magnetic bottle electron spectrometer, which uses a conical magnet of approximately 1 T at the point of interaction to create a divergent field that joins onto the homogeneous magnetic field of a few mT in a long solenoid, collects over 90% of all the nascent electrons. The 2 m long electron flight tube is terminated at the other end by a multi-channel plate detector with an efficiency of 50-60% for electrons with kinetic energies below 400 eV. This set-up is a more compact version of the original magnetic bottle electron coincidence apparatus described by Eland et al.^25^, and has a nominal resolving power of E_kin_/ΔE_kin_ ~ 50.

At BESSY-II, the set-up was connected to undulator beam line UE52/SGM where photon energies in the extreme ultraviolet and soft X-ray region can be selected as highly monochromatic beams. For the present experiments, a photon energy of 350.4 eV was used with the source in single bunch mode, providing light pulses with an inter-pulse spacing of 800.5 ns. The time interval between light pulses was increased to about 10 *μ*s by use of a mechanical chopper synchronised to the radio frequency tempo of the storage ring^26^. The flight times of the electrons were converted to kinetic energies using a calibration procedure that involved measurements of known photoelectron and Auger electron spectra of argon and krypton.

In the Gothenburg laboratory, a pulsed helium discharge lamp was used for ionisation, operated at a repetition rate of approximately 4 kHz. Photon energies of 21.2 eV (HeI*α*) and 40.8 eV (HeII*α*) were selected and focused into the interaction region using a monochromator based on an ellipsoidal grating. To calibrate the energy scale of the spectrometer in this energy range we used the valence single ionisation photoelectron spectrum of molecular oxygen.

### Computational details

The optimised geometry of the allene molecule was obtained within the DFT/B3LYP/aug-cc-pVTZ level of theory, as implemented in the *gaussian*16 package^27^, and it was kept fixed in the entire study. All ROHF and PT2 calculations were performed with the OpenMolcas software^28^. An investigation of the basis set dependence on the description of the singly and doubly ionized states was conducted. In Table 1 we show the ROHF results for all core level, valence photoelectron and core-valence states obtained with the ANO-RCC-VDZP, ANO-RCC-VTZP and ANO-RCC-VQZP basis sets. The results presented in the graphics are the ones obtained with the ANO-RCC-VQZP basis set, which is considered to be our main calculations. Results including PT2 are also shown. Comparing the results, we observe a systematic stabilisation of the states when increasing the basis set. For the core level spectrum presented in Supplementary Note 2, both C_*C*_ and C_*T*_ 1*s* singly ionized states show a stabilisation of more than 1.4 eV from the ANO-RCC-VDZP to the ANO-RCC-VQZP basis sets, while the relative energy between the states was not significantly altered. For the valence photoelectron spectrum also presented in Supplementary Note 2 we observe a similar feature, although the shifts are not larger than 0.15 eV. For all the 28 core-valence states, the obtained shift was typically about 1 eV from the ANO-RCC-VDZP to the ANO-RCC-VQZP basis sets. However, the stabilisation from the ANO-RCC-VTZP to the ANO-RCC-VQZP basis sets is about 0.2 - 0.4 eV for all states, which represents the expected convergence behaviour and which leads to the truly one-particle (ROHF) interpretation of the spectra.

Concerning the complete active space calculations we have chosen an active space in which each occupied orbital (fully HF occupied or strongly CAS occupied) is matched by an unoccupied orbital (HF unoccupied or weakly CAS occupied) and allowing for a complete redistribution of electrons in this space fulfilling spatial and spin symmetry constraints. This is a commonly used and well-balanced type of active space supported also by group theory and other arguments. For a low symmetry molecule with relatively many electrons such as core-valence ionized allene this type of CAS active gives a multitude of states (see computational section) many of which have the character of low-lying multiple excitations. In the two rightmost columns of Table 1, CASCI and CASCF energies are listed using this active space. We here find many main configuration states and states with multi-excitation characters mixed within a narrow energy region, where CASSCF with self-consistent optimisation also of the orbitals for each state gives further energy compression. Arguments from spectroscopic many-electron theory indicate that the states with leading multi-electron excitation configurations give low intensity and that only the main configuration eigenstates with leading single molecular orbital vacancy configurations (here pure core-valence molecular-orbital configurations) provide sizeable intensity. The square of the leading coefficients of these configurations (here core-2e or core-1e configurations) dictate their relative intensity in a simple model excluding the transition moments to the double electron continuum. In the CASCI spectrum, see Fig. 2, only transitions to these states are shown, modulated by the square of the leading coefficients, while the multi-electron states are neglected.

A multi-configuration calculation based on the CASCI methodology was conducted in order to clarify the quality of the one-particle approach of ROHF. We used the same ANO-RCC-VQZP basis set and the Hartree-Fock molecular orbitals obtained from the core ionized allene for constructing the configuration state functions. The active space was built with one core-orbital singly occupied, the lowest four valence orbitals of symmetry *a*_1_ and four orbitals of each *b*_1_ and *b*_2_ symmetries (2 occupied plus 2 unoccupied in the HF reference), resulting in 16 electrons in 16 orbitals. This setup gives rise to 7084 configuration state functions for the singlet states and 11262 for the triplet states. The results are displayed in Fig. 4b.

## Supplementary information

Supplementary Information

## Data Availability

The data sets generated during and/or analyzed during the current study are available from the corresponding authors on reasonable request.

## Code availability

The computer codes customised during the current study are available from the corresponding authors on reasonable request.
