# Supplementary material for: Symmetry breaking in core-valence double ionisation of allene
Source: Commun Chem. 2023 Jul 3;6:137. doi: 10.1038/s42004-023-00934-1 (PMC10317994; doi:10.1038/s42004-023-00934-1)
Supplement: Supplementary file 1 — Supplementary Information [file 42004_2023_934_MOESM1_ESM.pdf]

Supplementary Information
Symmetry breaking in core-valence double ionisation of allene

Veronica Ideböhn[1], Roberto Linguerri[2], Lucas M. Cornetta[3,4], Emelie Olsson[1], Måns Wallner[1], Richard J. Squibb[1], Rafael C. Couto[5], Leif Karlsson[3], Gunnar Nyman[6], Majdi Hochlaf[2], John H.D. Eland[7], Hans Ågren[3], and Raimund Feifel[1,*]

[1]University of Gothenburg, Department of Physics, Origovägen 6B, 412 58 Gothenburg, Sweden
[2]Université Gustave Eiffel, COSYS/IMSE, 5 Bd Descartes 77454, Champs sur Marne, France.
[3]Uppsala University, Department of Physics and Astronomy, Box 516, SE-751 20 Uppsala, Sweden
[4]Department of Applied Physics, Gleb Wataghin Institute of Physics, State University of Campinas, Campinas, Brazil
[5]Division of Theoretical Chemistry and Biology, School of Engineering Sciences in Chemistry, Biotechnology and Health, KTH Royal Institute of Technology, SE-106 91 Stockholm, Sweden
[6]University of Gothenburg, Department of Chemistry and Molecular Biology, 405 30 Gothenburg, Sweden
[7]Oxford University, Department of Chemistry, Physical and Theoretical Chemistry Laboratory, South Parks Road, Oxford OX1 3QZ, United Kingdom
[*]raimund.feifel@physics.gu.se

Supplementary Note 1: Experimental core-valence double ionisation spectra of CO_2

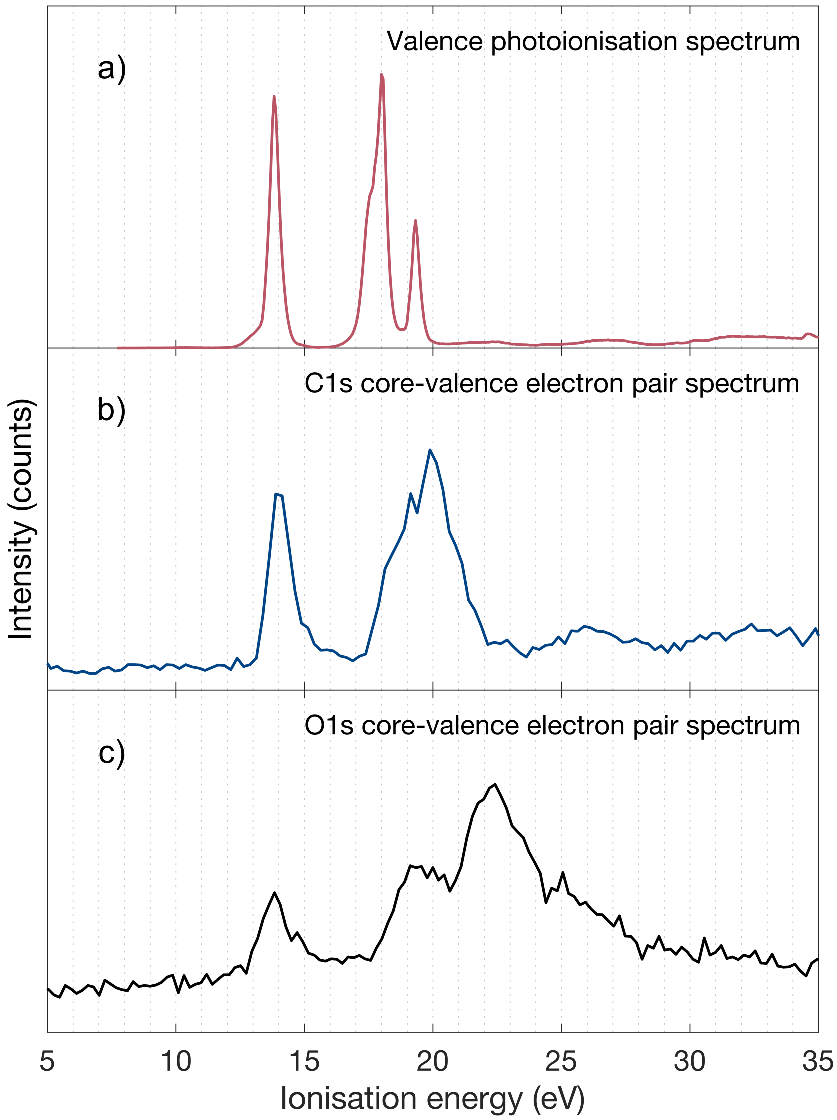

Supplementary Figure 1. Core-valence spectra of CO_2 obtained at photon energies of 360 eV for the C1s edge (panel b)) and 603 eV for the O1s edge (panel c)), respectively, in comparison with the valence photoelectron spectrum (panel a)) obtained at 40.81 eV photon energy. The core-valence spectra were shifted by 308.1 eV (panel b)) and 554.1 eV (panel c)), respectively, to line up with the lowest spectral feature of the valence photoelectron spectrum. Similar core-valence spectra of CO_2 have been published previously by some of us[1,2].

Supplementary Note 2: Experimental valence and carbon 1s ionisation spectra of allene in comparison with theoretical calculations

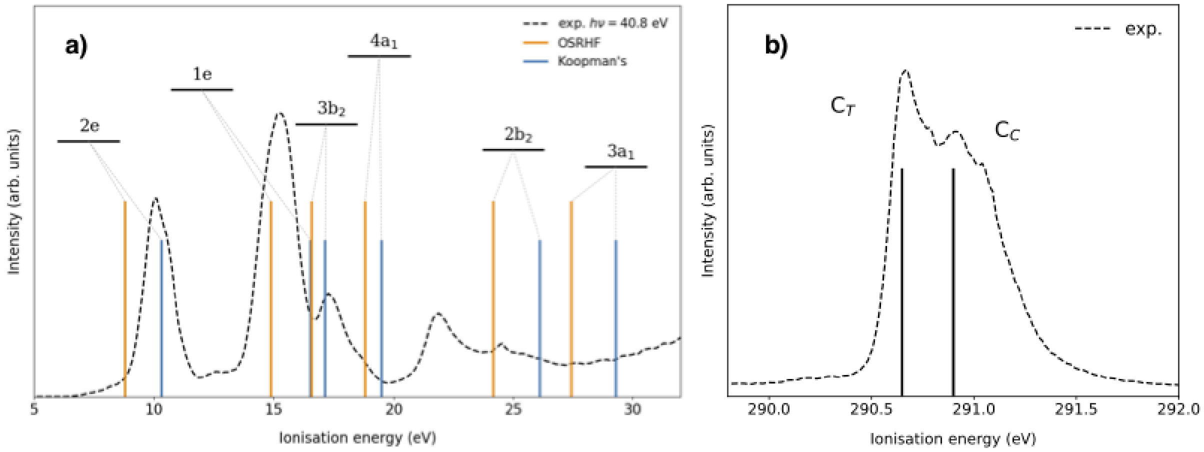

Supplementary Figure 2. Panel a): Theoretical calculations of the valence single ionisation energies of allene, in comparison to our experimental data. Panel b): Theoretical calculations of the single carbon 1*s* core-ionisation energies of allene, in comparison to the experimental data (taken with permission from the PhD thesis of Oksana Travnikova[3]). C_C and C_T denote the central and terminal carbon atoms, respectively.

Supplementary References

1. Eland, J. H. D. *et al.* Triple ionization of CO_2 by valence and inner shell photoionization. *J. Chem. Phys.* **135**, 134309, DOI: 10.1063/1.3643121 (2011). https://doi.org/10.1063/1.3643121.
2. Eland, J. H. D. & Feifel, R. *Double photoionisation spectra of molecules* (Oxford University Press, 2018).
3. Travnikova, O. *Structure and Dynamics of Core-Excited Species*. Ph.D. thesis, Acta Universitatis Upsaliensis (2008).
